# Enhancing Sorghum Growth: Influence of Arbuscular Mycorrhizal Fungi and Sorgoleone

**DOI:** 10.3390/microorganisms13020423

**Published:** 2025-02-15

**Authors:** Isabela Figueiredo de Oliveira, Maria Lúcia Ferreira Simeone, Ubiraci Gomes de Paula Lana, Cristiane de Carvalho Guimarães, Sylvia Morais de Sousa Tinôco

**Affiliations:** 1 Programa de Pós-Graduação em Bioengenharia, Universidade Federal de São João del-Rei, Praça Dom Helvécio 74, Fábricas, São João del-Rei 36301-160, Minas Gerais, Brazil; isabela.engquim@outlook.com; 2 Empresa Brasileira de Pesquisa Agropecuária, Embrapa Milho e Sorgo, Rod. MG 424 KM 65, Sete Lagoas 35701-970, Minas Gerais, Brazil; marialucia.simeone@embrapa.br (M.L.F.S.); ubiraci.lana@embrapa.br (U.G.d.P.L.); cristiane.guimaraes@embrapa.br (C.d.C.G.)

**Keywords:** AMF colonization, P uptake, plant growth, *Rhizophagus clarus*, *Sorghum bicolor*

## Abstract

The low availability of phosphorus (P) in soil is one of the main constraints on crop production. Plants have developed several strategies to increase P use efficiency, including modifications in root morphology, the exudation of different compounds, and associations with microorganisms such as arbuscular mycorrhizal fungi (AMF). This study aimed to investigate the effect of sorgoleone compound on AMF colonization and its subsequent impact on P uptake, rhizosphere microbiota, and sorghum growth. The experiment was conducted in a greenhouse using the sorghum genotype P9401, known for low sorgoleone production. Three doses of purified sorgoleone (20 μM, 40 μM, and 80 μM) were added to low-P soil and plants were harvested after 45 days. Treatments included inoculation with the arbuscular mycorrhizal fungi *Rhizophagus clarus* and a negative control without inoculum. The addition of 40 and 80 μM of sorgoleone did not significantly increase mycorrhization. However, treatment with 20 μM sorgoleone combined with *R. clarus* inoculation significantly increased total sorghum biomass by 1.6-fold (*p* ≤ 0.05) compared to the non-inoculated treatment. AMF inoculation influenced only AMF colonization and the fungal microbiota, without affecting the bacterial community, whereas sorgoleone showed no effect on either. The activities of acid and alkaline phosphatases in the rhizospheric soil did not differ significantly among the treatments. Furthermore, the sorghum genes *CYP71AM1*, associated with sorgoleone biosynthesis, and *Sb02g009880*, *Sb06g002560*, *Sb06g002540*, and *Sb03g029970* (related to phosphate transport induced by mycorrhiza) were significantly upregulated (*p* ≤ 0.05) in fine roots under these conditions. The 20 μM concentration of sorgoleone can enhance AMF colonization in sorghum and promote plant growth under low-P conditions, without significantly altering the microbiota.

## 1. Introduction

Arbuscular mycorrhizal fungi (AMF) are vital soil microorganisms that establish a non-pathogenic symbiotic relationship with the roots of most plant species. The symbiotic relationship between plants and AMF plays a crucial role in enhancing soil exploitation and improving nutrient acquisition efficiency, especially in cultivated crops such as maize [1,2], sorghum [3,4,5,6], and soybean [7,8]. The dynamics of mycorrhizal colonization and the environmental conditions influencing the symbiotic uptake of phosphate is essential for optimizing phosphorus (P) nutrition in crops [9,10]. Investigating the role of AMF in plant nutrition, especially in low-nutrient soils, holds significant potential for practical applications in agriculture. This is particularly important due to AMF’s capacity to alleviate both biotic and abiotic stresses on agriculturally important crops. AMF can induce morphological, biochemical, and physiological changes in plants, including alterations in gene expression, which can increase productivity. These effects are primarily attributed to their ability to enhance nutrient and water uptake through fungal mycelium extension [11]. AMF is estimated to increase the contact surface between plants and soil by up to 20 times, significantly enhancing phosphorus (P) absorption per unit of root length. This symbiotic relationship plays a crucial role in the growth and productivity of mycorrhizal plants compared to non-mycorrhizal ones. However, due to the complexity of mycorrhizal symbiosis, a greater diversity of elements is necessary to strengthen AMF community structure, which directly influences plant diversity and productivity [11]. Various AMF species contribute to crop productivity, including *Rhizophagus clarus* (formerly *Glomus clarum*), a member of the Glomeraceae family. This species has been shown to enhance nutrient uptake and plant growth across multiple agricultural systems and is distributed worldwide. Moreover, *Rhizophagus* can thrive and proliferate in semiarid ecosystems, even under conditions of limited water availability [12]. Notably, *R. clarus* produces larger spores compared to other species, such as *R. irregularis* and *R. intraradices* [13]. Due to these characteristics, *R. clarus* has garnered significant attention, particularly for its ability to grow symbiotically while utilizing myristate as a carbon and energy source [14,15].

Phosphorus deficiency is a major constraint on plant growth and crop yields in highly weathered soils, which are prevalent in tropical agricultural regions where food security remains a critical challenge. In these acidic soils, P becomes strongly bound to clay mineral surfaces, reducing its availability for plant uptake [16,17]. Plants such as sorghum have evolved morphological and physiological mechanisms to adapt to low P availability [18]. These adaptations include root system plasticity, the exudation of organic compounds, and symbiotic associations with microorganisms, such as AMF. Together, these strategies enhance the absorption and utilization of P nutrients by different crops [19,20]. While existing studies have primarily focused on the role of root system morphology in developing cultivars that are efficient in P acquisition [21,22,23,24,25], other mechanisms, such as associations with microorganisms [19,26,27], also contribute significantly to sorghum development in low-P soils.

The distinctive physical, chemical, and biological properties of molecules released in the rhizosphere, including sorgoleone [28], can shape the interactions between plants and microorganisms [4,5,26,29,30,31]. A study showed that a sorghum genotype releasing higher levels of sorgoleone per gram of dry root produced greater root and shoot biomass compared to lines producing medium or low levels of sorgoleone during the early growth stage [4]. Understanding the complex interactions between plant root exudates and the diverse soil microbial communities has the potential to offer valuable insights for improving crop sustainability and yield [4,5,11,26,31]. Under low-P conditions, the sorghum genotype P9401, inoculated with *Rhizophagus clarus* without sorgoleone, exhibited a 15% mycorrhizal colonization. However, with the addition of 20 µM sorgoleone, the mycorrhizal colonization increased significantly to 83%. This finding suggests that sorgoleone enhances mycorrhizal colonization, P content, and dry weight in P9401 [26]. However, the mechanism behind this enhancement remains unclear—whether increased sorgoleone directly stimulates AMF, improves P availability to enhance plant growth, or indirectly promotes root colonization through improved plant performance. The current study aimed to dissect the effect of sorgoleone on mycorrhizal colonization and rhizosphere microbiota and its role in improving P acquisition efficiency and sorghum growth.

## 2. Material and Methods

### 2.1. Sorgoleone Production and Purification

The sorghum genotype from Embrapa Maize and Sorghum Breeding Program (BR007B) was used to produce sorgoleone, as described by [26] for the greenhouse experiment. Briefly, after sorghum seed disinfection, they were placed on filter paper moistened with deionized water and kept in a germination chamber at a temperature of 25 °C for seven days in darkness. The sorgoleone extraction was performed by immersing roots in a solution of glacial acetic acid/dichloromethane solution 0.0025% (*v*/*v*) for 5 min. The resulting solution was filtered and the solvent was evaporated in a rotary evaporator at 40 °C. Subsequently, the roots were subjected to a forced air circulation oven at 65 °C until a constant weight was attained, allowing for the determination of dry mass.

**Figure 1:**
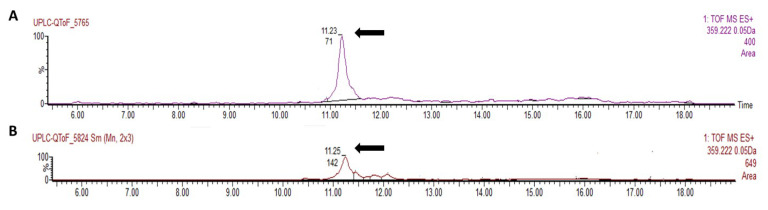
Chromatogram obtained using UPLC-QToF equipment for the sorgoleone standard at 500 ng mL^−1^ (**A**) and extracted from sorghum genotype BR007B (**B**) analyzed on a Waters Acquity UPLC BEH column in positive mode, with extracted ion *m*/*z* 359.2222 [M+H]+. The analysis was performed at Embrapa Agroindústria Tropical in Fortaleza, CE. The arrow indicates the retention time of the sorgoleone standard (11.23 min) and BR007B (11.25 min), followed by its relative area (71—standard and 142—BR007B).

The crude sorgoleone extract was applied to silica plates with hexane–isopropanol mixture (9:1 *v*/*v*), and the sorgoleone (RF = 0.35) band was carefully scraped off the plates and eluted with dichloromethane. The eluted sample was concentrated and the identification of sorgoleone was accomplished by comparing it with a standard retention time (Figure 1), as established by [32], utilizing High-Performance Liquid Chromatography (HPLC) with a Watters model Alliance 2695, PDA detector 2998, and a column Xbridge C18 (150 mm × 4.6 mm × 3.5 µm). During the chromatographic run (at 1.0 mL·min^−1^), the mobile phase consisted of acetonitrile (75% *v*/*v*) and an aqueous acetic acid solution (2.5% *v*/*v*), and the elution was monitored at a wavelength of 280 nm at room temperature (RT). The calibration curve (ranging from 0.015 to 0.125 mg mL^−1^), with a purified sorgoleone standard with a purity of 97.75%, was used to identify sorgoleone’s peak.

### 2.2. Inoculation of the P9401 Sorghum Genotype with Arbuscular Mycorrhizal Fungi and Sorgoleone Under Controlled Conditions

Sorghum P9401 genotype seeds [33] were germinated in paper rolls within a growth chamber and after four days were transplanted into pots filled with 2 kg of red latosol soil with a clayey texture (64% clay) from the Cerrado area at the Embrapa Maize and Sorghum Experimental Station in Sete Lagoas, Minas Gerais, Brazil (19°28′ S, 44°15′ W, at an altitude of 732 m above sea level), as described by [26,34]. The soil properties were as follows: Mehlich 1 extractable phosphorus (P) = 2.8 mg dm^−3^; potassium (K) = 44.3 mg dm^−3^; calcium (Ca) = 1.78 mg dm^−3^; magnesium (Mg) = 0.35 mg dm^−3^; cation exchange capacity (CEC) = 6.6 cmolc dm^−3^; potential acidity (H + Al) = 4.36 cmolc dm^−3^; base saturation = 33.9%; and organic matter (OM) = 3.49 g kg^−1^. To improve these properties, dolomite lime (2 g kg^−1^) and gypsum (0.5 g kg^−1^) were applied, and triple superphosphate was added to reach a final P concentration of 5 mg dm^−3^. The soil was left non-sterilized and retained its indigenous AMF community.

The experiment involved eight treatments distributed in a completely randomized design, consisting of 0 and 500 spores of mycorrhizal fungus *Rhizophagus clarus* supplemented with 0, 20, 40, and 80 µM sorgoleone. The inoculum was obtained from cultures of *R. clarus* (T.H. Nicolson and N.C. Schenck), C. Walker and A. Schüßler, using *Brachiaria decumbens* Stapf. Prain. as the host plant, maintained in a greenhouse for three months in 2000 mL pots. The roots of *B. decumbens* were collected, cut into small pieces, and mixed with the remaining soil. For spore counting, 1 g of inoculum was homogenized in water, sonicated, and filtered through sieves (500–38 μm) to remove organic material. The final fraction was centrifuged in a 20–60% sucrose gradient, and spores were collected from the supernatant on the 38 μm sieve, then counted on a grooved plate using an AXIO Zoom V16 stereomicroscope (Zeiss, North York, ON, Canada) [35,36]. The inoculation with *R. clarus* consisted of a mixture of 500 spores of *R. clarus* per plant with soil and grass root fragments. Each treatment involved two plants per pot, and three pots were employed per treatment. The sorgoleone solution, prepared in ethanol, was applied by adding 200 µL of each concentration directly into the planting hole of each seedling, applied once. Ethanol solvent was used as control [37].

Forty-five days after inoculation, the shoots were harvested, and the roots of both plants were carefully removed from the pots and thoroughly washed. Measurements were taken to assess root and shoot dry weight, P content, root morphology, and AMF colonization. The values represent the average of the two plants per pot. Roots were separated from the shoot, scanned, and analyzed with the software WinRHIZO v. 4.0 (Regent Systems Inc., Quebec City, QC, Canada) to measure traits related to root morphology, such as total root surface area (SA), surface area of roots with diameters between 0 and 1 mm (SA1), 1–2 mm (SA2), and larger than 2 mm (SA3) (cm^2^), total root length (L), and average root diameter (D) [38]. Roots and shoots were individually dried at 65 °C in a forced-air oven until a constant weight was achieved, allowing for the determination of the dry weight. For P content analysis, root and shoot tissues were ground using a Wiley mill and underwent nitric perchloric acid digestion at the Laboratório de Análises Ambientais e Agrícolas (LABRAS, Monte Carmelo, MG, Brazil) [39]. The P content was calculated by multiplying the dry weight of shoots and roots by their respective P concentrations.

### 2.3. Qualitative and Quantitative Assessments of Mycorrhizal Colonization

Fine fresh roots (~1 g) from the sorghum plants were placed in 70% (*v*/*v*) ethanol and stored in a refrigerator at 4 °C for 3 days. Then, the roots were washed with deionized water, clarified in a potassium hydroxide solution [KOH 10% (*w*/*v*)] overnight at RT, and washed and immersed in hydrochloric acid (HCl 0.3 M) for 30 min at RT. Following the acid treatment, the roots were stained with trypan blue solution [0.01% (*w*/*v*) trypan blue, 2.5% (*v*/*v*) acetic acid, and 50% (*v*/*v*) glycerol] for 30 min at RT.

The stained roots were transferred to conical tubes containing an acidified glycerol solution (1:1 glycerol and 0.3 M HCl). Mycorrhizal colonization was quantified using the gridline intersect method, as described by [40] with modifications from [26]. Total colonization was calculated by determining the proportion of intersections exhibiting specific fungal structures, such as vesicles or arbuscules. This assessment was conducted under an Axio Zoom V16 stereoscope (Zeiss, North York, ON, Canada) at 20-fold magnification, covering 100 intersection points per root sample.

Mycorrhizal colonization data were analyzed using analysis of variance (ANOVA), and treatment means were compared using Tukey’s test (*p* ≤ 0.05). Statistical analysis was conducted using Sisvar software, version 5.6 [41]. To ensure accurate statistical evaluation, mycorrhizal colonization percentages were normalized through an arcsine square root transformation prior to analysis.

### 2.4. Sorghum Gene Expression

Total RNA was isolated from fine root tissues of sorghum using the SV Total RNA Isolation System kit (Promega Corporation, Madison, WI, USA), according to the manufacturer’s instructions. Total RNA was used for cDNA synthesis using the High-Capacity cDNA Reverse Transcription kit (Applied Biosystems, Foster City, CA, USA), following the manufacturer’s instructions. Transcripts were quantified by quantitative real-time PCR (qPCR-RT), using SYBR Green technology with the ABI Prism 7500 Fast system (Applied Biosystems, Foster City, CA, USA). The relative gene expression (RQ) was calculated using the 2^−ΔΔCT^ method [42].

The expression profile of the genes related to sorgoleone biosynthesis—*CYP71AM1* [43], the phosphate transport induced by mycorrhiza—*Sb02g009880*, *Sb06g002560*, *Sb06g002540,* and *Sb03g029970* [44], and *AM3* and *RiEF* (Table 1) were assessed in the roots of the P9401 sorghum genotype with 0 and 20 µM sorgoleone inoculated and non-inoculated with *R. clarus*, as described above. We used *18S rRNA* as the reference gene (Table 1), and all reactions were performed in triplicate.

### 2.5. Microbial Diversity and Composition in the Sorghum Rhizosphere

#### 2.5.1. Soil DNA Extraction

Total DNA was extracted from 0.45 g of rhizospheric soil samples using the DNeasy PowerSoil Pro Kit (Qiagen, San Diego, CA, USA), according to the manufacturer’s instructions. DNA concentration was determined using a Nanodrop^®^ spectrophotometer (Thermo Fisher Scientific, Waltham, MA, USA), and the samples were adjusted to a final concentration of 5 ng μL^−1^.

#### 2.5.2. *16S* and *28S rRNA* Gene Amplification

For bacterial community analysis, the 16S rRNA gene was amplified using the fluorescence-labeled 8F-FAM primer (5′-AGAGTTGATCCTGGCTCAG-3′) [45] and the 1492R primer (5′-TACGGTACCTTGTACGACTT-3′) [46]. The PCR reaction mixture contained 2.5 ng of DNA, 1.0 μM of each primer, 1X reaction buffer, 3.12 mM MgCl_2_, 0.125 mM of each dNTP, and 1.25 U of Taq DNA polymerase (Invitrogen, Paisley, UK), in a final volume of 50 μL. Amplification was carried out with an initial denaturation at 94 °C for 3 min, followed by 25 cycles of 94 °C for 45 s, 55 °C for 45 s, and 72 °C for 2 min, with a final extension at 72 °C for 5 min.

For the analysis of the AMF community, a nested PCR approach was used to amplify the *28S rRNA* gene. The first PCR reaction utilized the primers LR1 (5′-GCATATCAATAAGCGGAGGA-3′) and FLR2 (5′-GTCGTTTAAAGCCATTACGTC-3′) [47], and the reaction mixture contained 2.5 ng of DNA, 1.0 μM of each primer, 1X reaction buffer, 2.5 mM MgCl_2_, 0.125 mM of each dNTP, 1.5 U of Taq DNA polymerase (Invitrogen, Paisley, UK), and 1 mM betaine, in a total volume of 50 μL. For the second PCR, 2.5 μL of the first reaction product was used, along with the FAM-labeled FLR3 primer (5′-TTGAAAGGGAAACGATTGAAGT-3′) and the HEX-labeled FLR4 primer (5′-TACGTCAACATCCTTAACGAA-3′) [48], at a final concentration of 1.0 μM each, 1X reaction buffer, 2.5 mM MgCl_2_, 0.125 mM of each dNTP, and 1.5 U of Taq DNA polymerase (Invitrogen, Paisley, UK), in 50 μL. The amplification of AMF was performed with an initial denaturation at 95 °C for 5 min, followed by 35 cycles of 94 °C for 1 min, 60 °C for 1 min, 72 °C for 1 min, and a final extension at 72 °C for 10 min.

A 1 μL aliquot of the PCR products was stained with GelRed (Biotium, Fremont, CA, USA) and analyzed using 1% (*w*/*v*) agarose gel electrophoresis, with a 1 Kb Plus DNA Ladder (Life Technologies, Carlsbad, CA, USA). The amplified DNA was visualized under ultraviolet light using a transilluminator and photographed with the L-PIX Image EX system (Loccus Biotecnologia, Cotia, SP, Brazil).

#### 2.5.3. Terminal Restriction Fragment Length Polymorphism (T-RFLP) Profile Analysis

The amplified fragments were digested with the restriction enzymes *Alu*I, *Hae*III, and *Hpa*II (Invitrogen, Carlsbad, CA, USA). To prepare the DNA fragments for analysis, 2 μL of the digestion product was mixed with 9.8 μL of deionized formamide (Applied Biosystems, Foster City, CA, USA) and 0.2 μL of the ROX 500 standard (Applied Biosystems, Foster City, CA, USA). The PCR-digested products were then resolved via capillary electrophoresis using the Genetic Analyzer 3500XL (Applied Biosystems, Foster City, CA, USA), with data analysis performed using GeneMapper 5.0 software (Applied Biosystems, Foster City, CA, USA). Terminal Restriction Fragment (T-RF) peaks ranging from 30 to 500 bp and exhibiting fluorescence intensities greater than 40 fluorescence units (peak height) were included in the profile analysis.

The T-Rex program was used to align the samples and generate consensus profiles from two parallel runs of each sample. Only T-RFs with a relative abundance of ≥1% (calculated as the individual peak area divided by the total peak area) were considered for data generation. The relative abundance of microbial species was determined based on the average T-RF size values resulting from digestion with the restriction enzymes. To calculate the similarity between fragment sizes, Past software [49] was used. The diversity profiles of bacteria and AMF were then evaluated using non-metric multidimensional scaling (NMDS), based on the Bray–Curtis distance matrix. To assess whether the sample groups exhibited significantly different means, a one-way similarity analysis test (ANOSIM) was conducted, with a significance level of 95% (*p* ≤ 0.05).

#### 2.5.4. Bacterial Community Identification

The T-RFs were compared by aligning the observed fragment lengths with the predicted lengths generated from the three restriction enzymes. This alignment was performed using the Microbial Community Analysis III (MiCA 3) tool (http://mica.ibest.uidaho.edu/, accessed on 10 January 2023). For taxonomic classification, data were obtained from NCBI using the Taxonomy Status tool (https://www.ncbi.nlm.nih.gov/Taxonomy/TaxIdentifier/tax_identifier.cgi, accessed on 10 February 2023).

### 2.6. Phosphatases Activities

The methods employed for assessing phosphatase enzyme activities were detailed by [50]. The procedures encompass the extraction and quantitative determination of micrograms of p-nitrophenol released during soil incubation with either p-nitrophenyl phosphate or bis-p-nitrophenyl phosphate. This incubation occurs in a modified universal buffer adjusted to pH 6.5 and 11 for acid and alkaline phosphatase, respectively. The enzymatic reactions were halted by adding CaCl_2_ and NaOH, and the resulting solutions were subjected to centrifugation at 6000× *g* for 5 min. Subsequently, the supernatant was utilized for colorimetric measurements at 400 nm. Enzyme activities (µg pnitrofenol h^−1^ g^−1^ dry soil) were assessed in triplicate for each rhizosphere soil sample.

### 2.7. Data Statistical Analysis

The analysis of variance (ANOVA) for root morphology traits, dry weight, P content, mycorrhizal colonization, gene expression, and soil enzymatic activity was conducted using the “ExpDes.pt” package [51] within the R software [52]. The Tukey test was employed for statistical comparisons of means, with a significance level of 95% (*p* ≤ 0.05). Principal Component Analysis (PCA) was conducted with the “factoextra” package [53] in the R software.

For the relative abundance analysis of bacterial community phyla resulting from taxonomic identification via T-RFLP analysis, ggplot2 [54] and reshape2 [55] packages in the R software were utilized. ANOVA was conducted for the evaluated treatments using the “ExpDes.pt” package [51], and for statistical comparisons of means, the LSD test was employed, with a significance level of 95% (*p* ≤ 0.05).

## 3. Results

### 3.1. The Concentration of 20 µM Sorgoleone Promotes Increased Mycorrhization, P Content, and Plant Biomass

The analysis of variance for root morphology, plant biomass, P content, and AMF colonization revealed significant variability in the traits (Table 2). A significant increase in plant length, total surface area, plant biomass, and shoot, root, and total P content was observed in the presence of the fungus *R. clarus* with 20 µM sorgoleone (Table 2). However, higher concentrations of sorgoleone (40 and 80 µM) did not lead to a significant increase in mycorrhization (Table 2), suggesting that the response to sorgoleone addition is dose dependent.

**Figure 2:**
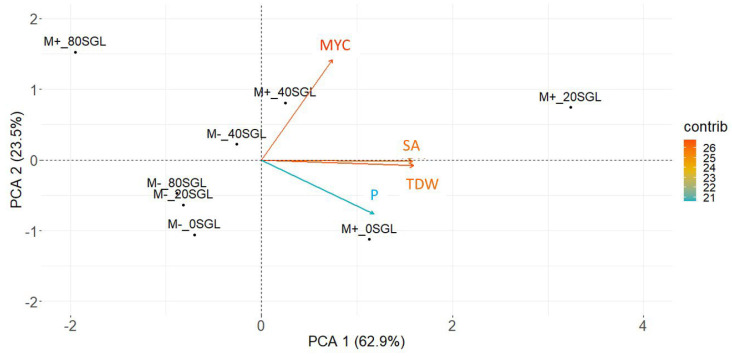
Principal component analysis (PCA) for sorghum plants grown in a greenhouse under low-phosphorus soil supplemented with 0, 20, 40, and 80 µM of sorgoleone, and with (M+) and without (M−) inoculation of *Rhizophagus clarus*. SA: total surface area; TDW: total dry weight; P: total P content; and MYC: mycorrhization.

In the PCA, the first and second principal components (PC1 and PC2) explained 62.9% and 23.5%, respectively, totaling 86.4% of the variation within treatments (Figure 2). PCA was able to differentiate treatments based on the selected traits. In general, treatments with AMF (M+) were in the right quadrants, while those without AMF (M−) inoculation were on the left. The treatments with 20 and 40 µM of sorgoleone were in the upper quadrant and without sorgoleone in the lower quadrant. Considering the results (Table 2) and comparing them with their position on the scatterplot, we observed that 20 µM of sorgoleone presented higher mycorrhization, dry weight, and total root surface than the other treatments (Figure 2).

### 3.2. Overexpression of Genes Related to Sorgoleone Pathway and Mycorrhization

**Figure 3:**
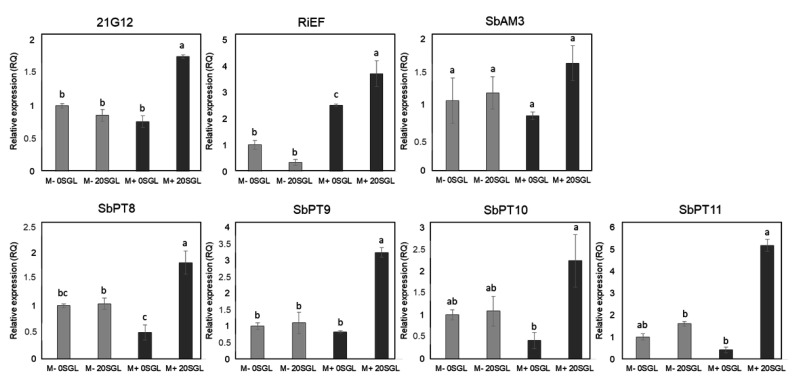
Relative expression of genes of *CYP71AM1* (*21G12*), *RiEF*, *AM3* (*SbAM3*), *Sb02g009880* (*SbPT8*), *Sb06g002560* (*SbPT9*), *Sb06g002540* (*SbPT10*), and *Sb03g029970* (*SbPT11*) measured in the roots of sorghum plants grown in a greenhouse under low-P conditions with 0 (0SGL) and 20 µM (20SGL) of sorgoleone, with (M+) and without inoculation (M−) of *Rhizophagus clarus* after 45 days. Error bars are standard error of the mean (SEM) of three biological replications. The bars with the same letter do not differ by the Tukey test (*p* < 0.05).

We selected 0 and 20 µM sorgoleone treatments for further examination based on the morphophysiological analysis. The gene expression results revealed a significant upregulation (*p ≤* 0.05) of the *CYP71AM1* (*21G12*) gene, associated with sorgoleone biosynthesis, and *Sb02g009880* (*SbPT8*), *Sb06g002560* (*SbPT9*), *Sb06g002540* (*SbPT10*), and *Sb03g029970* (*SbPT11*) genes, linked to phosphate transport induced by mycorrhiza and RiEF, which is a marker for AMF inoculation with sorgoleone and AMF inoculation (Figure 3). There was an increased expression, although not significant, of the *AM3* (*SbAM3*) gene, which served as marker for AMF colonization (Figure 3).

### 3.3. Effect of Treatments on Soil Microbiota

**Figure 4:**
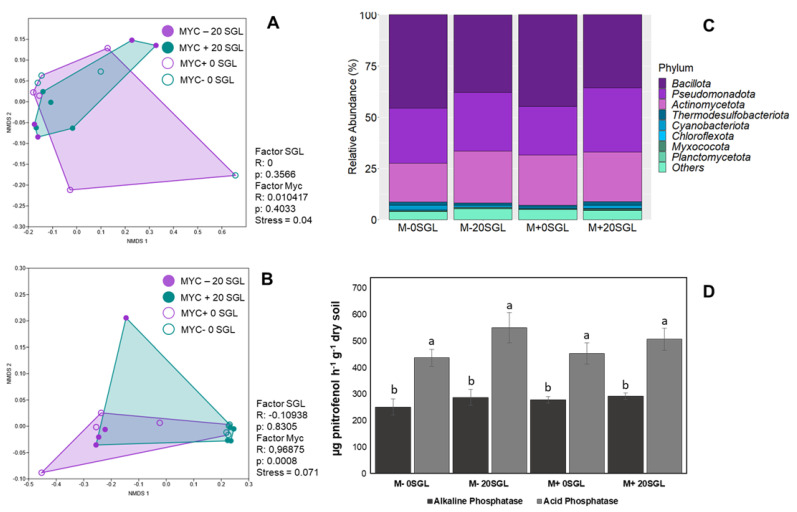
Profile based on non-metric multidimensional scaling (NMDS) using the Bray–Curtis distance matrix to analyze the genetic diversity of the bacterial community (**A**) and arbuscular mycorrhizal fungi (**B**), relative abundance of the phyla of the bacterial community (**C**) and alkaline and acid phosphatase activities (**D**) from rhizosphere soil of sorghum grown in a greenhouse under low P with and without inoculation of *Rhizophagus clarus* and supplemented with 0 and 20 µM of sorgoleone. Error bars are standard error of the mean (SEM) of three biological replications. The bars with the same letter do not differ by the Tukey test (*p* < 0.05).

The non-metric multidimensional scaling (NMDS) analysis indicated that the bacterial community remained unchanged with the introduction of AMF and sorgoleone (Figure 4A). However, a significant difference was detected in the genetic diversity profile of the AMF community, indicating dissimilarity between treatments with and without *R. clarus* inoculation, but not sorgoleone (Figure 4B). Moreover, no statistically significant differences were detected in the relative abundance of bacterial phyla across the treatments (including both sorgoleone and AMF inoculation) (Figure 4C). The most abundant phylum in the four treatments were Bacillota, Pseudomonadota, and Actinomycetota (Figure 4C). There was no significant increase in the enzymatic activity of acid and alkaline phosphatases (Figure 4D).

## 4. Discussion

In a previous study by [26], low concentrations of sorgoleone (5, 10, and 20 μM) were tested in the sorghum genotype P9401. This genotype is known for its resistance to *Striga*, an obligate parasitic plant prevalent in regions of Africa and Asia, which commonly infests various crops, including sorghum [56,57,58,59]. Additionally, P9401 exhibits low root exudation, resulting in the minimal stimulation of *Striga* seed germination [58,59]. In the present study, we investigated the effects of higher sorgoleone concentrations, both with and without AMF, on sorghum growth, rhizosphere microbiota, the expression of genes related to the sorgoleone pathway, and AMF. Our results confirmed that 20 µM of sorgoleone improved mycorrhization, plant biomass, and P content in mycorrhizal plants compared to non-mycorrhizal plants cultivated under low P. The response to sorgoleone appears to have an optimal dose, as the plant becomes unresponsive at lower doses [26], while higher doses exceeding 20 µM do not inhibit root and plant growth (Table 2). It is still necessary to understand the relationship between the various genes and enzymes of the pathway and their interactions with different plant hormones, especially auxin, as has been advanced for strigolactone [60]. Our work (Table 2) corroborated the findings that sorghum inoculation with AMF enhances plant growth, leading to significant increases in shoot height and root length [61], and dry weight [62]. Furthermore, AMF inoculation contributes to greater nutrient absorption [4,5,63] and higher grain productivity [6].

It is crucial to recognize the increase in root surface area facilitated by hyphae of AMF, as observed in this study (Table 2). The expanded volume and extension of soil explored by fungal hyphae are instrumental in enhancing nutrient absorption, particularly P. Hyphae, which can extend beyond the nutrient-depleted zone that forms around absorbing roots. In this zone, there is a reduction in the concentration, especially of P, which has a slower transport rate in the soil solution and limited availability compared to the plant’s demand. While roots rapidly absorb phosphate ions in their proximity, the soil solution struggles to balance the concentration in this region due to slow transport and low P concentration. Thus, AMF plays a crucial role in P absorption by extending beyond the depletion zone and exploring a larger soil volume in their search of nutrients [64].

Although sorghum varieties with higher sorgoleone exudation tend to develop a denser mycorrhizal network in rhizosphere soils [4,12,65], mycorrhizal colonization appears to be suppressed with more than 20 μM sorgoleone. The plant roots incur an energy cost with the exudation of signaling compounds for spore germination and branching of fungal hyphae in the rhizosphere during pre-symbiotic recognition. Additionally, in plant–mycorrhizal symbiosis, both symbionts are capable of detecting variations in the resources provided by each other, allowing them to adjust their resource allocation to strike a balance between costs and benefits under varying P conditions [15,66]. Therefore, it is conceivable that 20 µM represents the optimal concentration for plant–AMF signaling, consistent with the sensitivity expected for a plant signaling substance [67]. Strigolactones, which are phytohormones known to enhance plant growth under abiotic stress, present the optimal concentration for mitigating drought stress in maize [68].

Furthermore, in a greenhouse, no alteration was observed in the bacterial and fungal communities of rhizosphere soil with the addition of sorgoleone (Figure 4). We observed a higher relative abundance of taxa associated with efficient carbon mineralization, including Bacillota, Pseudomonadota, and Actinomycetota, along with an underrepresentation of phyla negatively correlated with carbon mineralization (Figure 4). This increased microbial carrying capacity likely reflects the enhanced nutrient flux in absorptive fine roots and corresponds to the proliferation of taxa known for their copiotrophic lifestyles. Nevertheless, a significant difference was observed in the genetic diversity profile of the AMF community, emphasizing the dissimilarity between treatments with and without *R. clarus* inoculation. Some studies indicate that sorgoleone does influence the dynamics of the microbial community structure in rhizosphere soils of field-grown sorghum [5,27], highlighting the necessity for further investigation. It is worth noting that sorghum’s field performance, especially its response to AMF colonization in terms of improved growth and/or P uptake, may vary depending on the genotype, adding another variable to consider. The genotype significantly influences the microbial communities in the rhizosphere and the regulation of ecological services provided by plant-associated microbes in interaction with the plant. The composition of rhizosphere communities is predominantly influenced by the plant species they are associated with [69], primarily through the selection of microbes capable of utilizing the carbon source profile released by the roots [70]. Although indigenous soil was used, the experiment was conducted under controlled conditions and within a limited time frame, which may have constrained the modulation of the bacterial community. The introduction of AMF was expected to induce a more pronounced shift in the microbiota, which is consistent with our findings. Alternatively, it is possible that changes in fungal communities occur first, with subsequent alterations in the bacterial communities developing over time. To validate this hypothesis, more extensive, long-term studies, including field experiments, are needed.

The analogy also extends to the observation that no significant difference was noted in the activity of acid and alkaline phosphatases (Figure 4D). The activity of acid phosphatase was higher than that of alkaline phosphatase, which is expected since acid phosphatase is produced by both plants and microorganisms, while alkaline phosphatase is primarily of microbial origin. These enzymes are linked to P remobilization in plants, and higher enzyme activity is typically associated with low cellular phosphate levels [71,72,73]. Since AMF promotes increased phosphate uptake, there is no incentive for plant roots to invest in secreting phosphatases. Hyphae prove to be much more efficient in creating a plant–soil contact surface for nutrient capture, with substantially lower carbon (energy) expenditure than roots. Consequently, it is more efficient for the plant to invest in mycorrhiza than in root production.

The findings of our study also revealed an upregulation in the expression of a genes involved in the sorgoleone biosynthesis pathway and phosphate transporters induced by AMF (Figure 3). P uptake in plants involves the transport of Pi (inorganic orthophosphate) across the plasma membrane into the root symplasm, mediated by Pi/H+ symporters from the *Pht*1 gene family [74,75]. *Pht*1 genes are predominantly expressed in rhizodermal and cortical cells, supporting the “direct pathway” of Pi uptake from the soil. In AMF symbiosis, an additional “mycorrhizal pathway” is utilized, where Pi is absorbed by fungal extraradical hyphae, translocated to the roots, and released into the periarbuscular space [76]. Pi is subsequently taken up by AMF-specific *Pht*1 transporters, which are classified into subfamilies I and III [70]. Subfamily I transporters are expressed in cortical cells containing arbuscules, while subfamily III transporters are generally induced in roots, but are particularly concentrated in cortical cells during AM symbiosis [77,78]. Therefore, the overexpression of *SbPT*8, *SbPT*9, *SbPT*10, and *SbPT*11 genes, which are essential for mycorrhizal Pi uptake [79], provides strong evidence that sorgoleone and AMF together enhance AMF colonization. The biosynthesis of sorgoleone involves several key enzymatic steps. It begins with the production of an alkylresorcinol intermediate (SbARS2) from fatty acyl-CoA, catalyzed by *Sorghum bicolor* fatty acid desaturases (SbDES2 and SbDES3). This intermediate is methylated by O-methyltransferase SbOMT3. The final step involves the cytochrome P450 enzyme (CYP71AM1), which converts 5-pentadecatrienyl resorcinol-3-methyl ether into dihydrosorgoleone. Released into the soil, dihydrosorgoleone auto-oxidizes to form the more stable benzoquinone, sorgoleone [43,80]. The overexpression of CYP71AM1 provides evidence that sorgoleone production was induced by the presence of AMF and exogenous sorgoleone. Additionally, the overexpression of the fungal *RiEF*1 reference gene further supports the observed increase in AMF colonization in the presence of both AMF and sorgoleone (Table 2). While the importance of root exudation for effective symbiosis between plants and AMF has been established across various plant species such as maize, soybean, and sorghum [3,4,5,9,26,31,81,82,83], this study reaffirms sorgoleone’s role as a signaling mechanism for symbiosis with AMF and a subsequent increase in P uptake.

Despite the highly regulated and dynamic nature of mycorrhizal colonization, the factors influencing sorgoleone’s mode of action, bioactive concentration, persistence, and release in the rhizosphere, as well as its absorption and translocation, remain incompletely understood [5,11,43,84,85]. Further studies are warranted to explore the effects of bioactive molecules on plants, given that the root exudation of fungal signaling compounds is a fundamental aspect of the symbiotic response [11]. This understanding not only sheds light on the regulation of sorgoleone production, but also offers opportunities to manipulate crop levels to enhance agricultural productivity.

## 5. Conclusions

Since its discovery, sorgoleone has been recognized as a key secondary metabolite with diverse applications. Understanding the hormetic effects of this plant-released phytotoxin is essential for unraveling its role in microorganism–plant–soil interactions. A concentration of 20 μM sorgoleone has been shown to positively impact sorghum mycorrhization, leading to increased plant dry weight and P content. Additionally, the upregulation of sorghum genes involved in the sorgoleone biosynthesis pathway and phosphate transporters, induced by AMF, highlights its role as a signaling molecule in AMF symbiosis, enhancing phosphorus uptake. Investigating genes and functional genomics is critical for understanding the transcriptional regulation of genes and regulatory factors involved in sorgoleone biosynthesis and AMF colonization. Ongoing research in this area promises to reveal the complexities of sorgoleone’s biosynthesis and function, especially related to AMF, with significant potential for applications in agriculture and ecology.

## Figures and Tables

**Table 1:** Primers used for gene expression analysis in sorghum roots by quantitative real-time PCR (q-PCR).

Description	Gene	Primer	Sequence (5′–3′)
Cytochrome P450 enzyme involved in biosynthesis of sorgoleone	*CYP71AM1*	*21G12*	Fwd—AAGATCCAAGGCTACCATGTGC Rev—AACGTTGGCGACGACTATTG
AMF colonization	*AM3*	*SbAM3*	Fwd—GGCAGCAACAAGGCTAATTC Rev—ACCCTTGTGACGGAGAACAC
*Rhizophagus irregulares* Elongation factor	*RiEF*	*RiEF*	Fwd—TGTTGCTTTCGTCCCAATATC Rev—GGTTTATCGGTAGGTCGAG
AMF-induced Pi transporter	*Sb02g009880*	*SbPT8*	Fwd—GCAGCGAGGCCAATGAGACT Rev—TTGGCTCCGGTAGGAAGCAG
AMF-induced Pi transporter	*Sb06g002560*	*SbPT9*	Fwd—GAGGACGAGCCGTTCAAGAG Rev—CGCGACGGAGAAGAAGTACC
AMF-induced Pi transporter	*Sb06g002540*	*SbPT10*	Fwd—CACCATGTGCTGGTTACTTC Rev—GATAATCGCCTGAGTACGTG
AMF-induced Pi transporter	*Sb03g029970*	*SbPT11*	Fwd—CGTGGTTCCTTCTGGACATA Rev—TCTCGAACACCTCCTTGAGT
Endogenous control	*18S rRNA*	*18S*	Fwd—AATCCCTTAACGAGGATCCATTG Rev—CGCTATTGGAGCTGGAATTACC

**Table 2:** Analysis of Variance (ANOVA) for morphophysiological traits and mycorrhization of the sorghum genotype P9401 cultivated with different concentrations of sorgoleone, in the presence (Myc+) and absence (Myc−) of the arbuscular mycorrhizal fungus (AMF) *Rhizophagus clarus*, under low phosphorus (P) in greenhouse conditions.

Traits	AMF	Sorgoleone (µM)			
		0	20	40	80
L (cm)	Myc+	2472.57 ± 268.16 ABa	3360.02 ± 579.36 Aa	2930.90 ± 34.44 Aa	1355.48 ± 439.70 Ba
	Myc−	1769.02 ± 127.39 Aa	2082.23 ± 119.22 Ab	2830.16 ± 600.79 Aa	1683.63 ± 128.02 Aa
SA (cm^2^)	Myc+	557.71 ± 20.88 Aa	636.14 ± 96.29 Aa	484.39 ± 52.93 Aa	214.84 ± 59.21 Ba
	Myc−	347.40 ± 35.85 Ab	360.20 ± 49.57 Ab	385.97 ± 93.79 Aa	216.76 ± 31.95 Aa
D (mm)	Myc+	0.370 ± 0.04 Aba	0.376 ± 0.07 ABa	0.311 ± 0.03 Ba	0.509 ± 0.02 Aa
	Myc−	0.313 ± 0.02 Aa	0.272 ± 0.02 Aa	0.388 ± 0.08 Aa	0.301 ± 0.12 Ab
SA1 (cm^2^)	Myc+	195.47 ± 22.23 ABa	268.42 ± 54.08 Aa	233.40 ± 7.17 Aa	115.03 ± 33.94 Ba
	Myc−	136.23 ± 10.80 Aa	162.21 ± 1.96 Ab	218.70 ± 49.54 Aa	135.95 ± 10.20 Aa
SA2 (cm^2^)	Myc+	108.96 ± 11.55 ABa	129.58 ± 20.86 Aa	96.15 ± 13.68 ABa	56.19 ± 16.30 Ba
	Myc−	68.68 ± 4.82 Ab	74.89 ± 11.64 Ab	70.73 ± 17.11 Aa	38.50 ± 9.90 Aa
SA3 (cm^2^)	Myc+	163.04 ± 22.14 Aa	149.99 ± 13.34 Aa	90.15 ± 29.53 ABa	21.00 ± 1.42 Ba
	Myc−	92.33 ± 21.30 Ab	75.36 ± 19.38 Ab	45.21 ± 17.78 Aa	15.78 ± 7.29 Aa
RDW (g)	Myc+	0.87 ± 0.09 Aa	0.92 ± 0.11 Aa	0.62 ± 0.15 Aa	0.19 ± 0.00 Aa
	Myc−	0.44 ± 0.11 Aa	0.46 ± 0.10 Aa	0.91 ± 0.73 Aa	0.08 ± 0.10 Aa
SDW (g)	Myc+	1.36 ± 0.16 Aba	1.72 ± 0.43 Aa	1.37 ± 0.27 ABa	0.60 ± 0.10 Ba
	Myc−	1.20 ± 0.18 Aa	0.84 ± 0.21 Ab	0.75 ± 0.12 Ab	1.11 ± 1.04 Aa
TDW (g)	Myc+	2.23 ± 019 Aba	2.64 ± 0.53 Aa	1.99 ± 0.42 ABa	0.79 ± 0.10 Ba
	Myc−	1.64 ± 0.27 Aa	1.30 ± 0.28 Ab	1.66 ± 0.75 Aa	1.19 ± 1.15 Aa
MYC (%)	Myc+	26.00 ± 1.82 Ba	67.25 ± 2.99 Aa	39.50 ± 4.58 ABa	20.00 ± 4.24 ABa
	Myc−	17.75 ± 1.19 Ba	24.25 ± 1.72 Bb	28.25 ± 2.27 Aa	18.50 ± 2.12 ABa
RPCont (g)	Myc+	0.45 ± 0.03 Aa	0.51 ± 0.06 Aa	0.36 ± 0.08 Aa	0.03 ± 0.00 Aa
	Myc−	0.24 ± 0.06 Aa	0.25 ± 0.05 Ab	0.43 ± 0.37 Aa	0.06 ± 0.08 Aa
SPCont (g)	Myc+	1.00 ± 0.09 Aa	1.56 ± 0.44 Aa	1.20 ± 0.08 Aa	0.83 ± 0.35 Aa
	Myc−	1.06 ± 0.14 Aa	0.83 ± 1.16 Ab	1.10 ± 0.25 Aa	0.72 ± 1.75 Aa
TPCont (g)	Myc+	1.46 ± 0.10 Aa	2.07 ± 0.44 Aa	1.57 ± 0.16 Aa	0.87 ± 0.35 Aa
	Myc−	1.30 ± 0.21 Aa	1.08 ± 0.18 Ab	1.54 ± 0.36 Aa	1.78 ± 1.84 Aa
Soil_PCont (g)	Myc+	13.50 ± 2.95 Aa	11.27 ± 1.56 Aa	13.45 ± 2.28 Aa	8.60 ± 2.54 Aa
	Myc−	12.65 ± 2.50 Aa	11.15 ± 1.18 Aa	15.95 ± 6.77 Aa	10.50 ± 0.56 Aa

Means followed by the same capital letters indicate non-significant differences between sorgoleone concentrations, and identical lower-case letters indicate non-significant differences between non-inoculated and inoculated with AMF, using Tukey’s test at 5% probability. Standard error of the mean (SEM) of three biological replications. Total root length (L), total root surface (SA), average root diameter (D), surface area of roots with diameters between 0 and 1 (SA1), 1 and 2 (SA2), and 2 to 4, 5 mm (SA3), root, shoot, and total dry weight (RDW, SDW, and TDW), quantitative analysis of AMF colonization (MYC) and root, shoot, total, and soil P content (RPCont, SPCont, TPCont, and Soil_PCont).

## Data Availability

The original contributions presented in the study are included in the article, further inquiries can be directed to the corresponding author.
